# Testosterone boosts physical activity in male mice via dopaminergic pathways

**DOI:** 10.1038/s41598-017-19104-0

**Published:** 2018-01-17

**Authors:** Ferran Jardí, Michaël R. Laurent, Nari Kim, Rougin Khalil, Dimitri De Bundel, Ann Van Eeckhaut, Lawrence Van Helleputte, Ludo Deboel, Vanessa Dubois, Dieter Schollaert, Brigitte Decallonne, Geert Carmeliet, Ludo Van den Bosch, Rudi D’Hooge, Frank Claessens, Dirk Vanderschueren

**Affiliations:** 1 Clinical and Experimental Endocrinology, Department of Clinical and Experimental Medicine, KU Leuven, Herestraat 49 PO box 902, 3000 Leuven, Belgium; 2 Molecular Endocrinology Laboratory, Department of Cellular and Molecular Medicine, KU Leuven, Herestraat 49 PO box 901, 3000 Leuven, Belgium; 3 Gerontology and Geriatrics, Department of Clinical and Experimental Medicine, KU Leuven, Herestraat 49 PO box, 7003 Leuven, Belgium; 4 Department of Pharmaceutical Chemistry and Drug Analysis, VUB, Laarbeeklaan 103, 1090 Brussels, Belgium; 5 Laboratory of Neurobiology, VIB Center for Brain and Disease Research and KU Leuven, Herestraat 49 PO box 602, 3000 Leuven, Belgium; 6 Laboratory of Biological Psychology, Faculty of Psychology and Educational Sciences, KU Leuven, Tiensestraat 102, 3000 Leuven, Belgium; 7 Present Address: INSERM UMR1011, University of Lille and Institut Pasteur de Lille, Lille, France

## Abstract

Low testosterone (T) in men, especially its free fraction, has been associated with loss of energy. In accordance, orchidectomy (ORX) in rodents results in decreased physical activity. Still, the mechanisms through which T stimulates activity remain mostly obscure. Here, we studied voluntary wheel running behavior in three different mouse models of androgen deficiency: ORX, androgen receptor (AR) knock-out (ARKO) and sex hormone binding globulin (SHBG)-transgenic mice, a novel mouse model of “low free T”. Our results clearly show a fast and dramatic action of T stimulating wheel running, which is not explained by its action on muscle, as evidenced by neuromuscular studies and in a muscle-specific conditional ARKO mouse model. The action of T occurs via its free fraction, as shown by the results in SHBG-transgenic mice, and it implies both androgenic and estrogenic pathways. Both gene expression and functional studies indicate that T modulates the *in vivo* sensitivity to dopamine (DA) agonists. Furthermore, the restoration of wheel running by T is inhibited by treatment with DA antagonists. These findings reveal that the free fraction of T, both via AR and indirectly through aromatization into estrogens, stimulates physical activity behavior in male mice by acting on central DA pathways.

## Introduction

Physical inactivity has emerged as a global epidemic. Less than 50% of the adult Western population meets the minimum levels of recommended moderate-to-vigorous exercise, whilst spending 60% of their time in sedentary pursuits^1,2^. The lack of regular exercise is estimated to cause 6% of the burden of disease from coronary heart disease, 7% of type 2 diabetes and 10% of both breast and colon cancer^3^, and accounts for 1.5–3.0% of direct healthcare costs^4^. It is of no surprise, therefore, that implementing regular exercise on a population level has become a key priority in public health policy.

Over the past several years, new insights have revealed the existence of a neurophysiological basis regulating the motivation to engage in regular physical exercise. Locomotion is a well-established sexually dimorphic behavior in rodents^5^ that responds to androgens and estrogens^6,7^. Diminished self-reported physical activity was associated to low testosterone (T) levels in a cohort of 1954 German men^8^. Still, the association between sex steroids and physical activity in humans, as assessed by objective measures, has not been studied so far.

With ageing, serum total T concentrations gradually decrease in men^9^. In parallel, there is an increase in the concentrations of sex hormone-binding globulin (SHBG)^9^, a high-affinity binding protein for androgens and estrogens. This results in a more pronounced reduction of bioavailable or free T levels as men age^9^. We recently showed that free T concentrations correlated better than total T with androgen deficiency–related symptoms and signs in men with late-onset hypogonadism^10^. Despite controversy surrounding the diagnostic criteria and treatment of so-called late-onset male hypogonadism, sales of prescription T replacement drugs in US topped $1.8 billion in 2011^11^. Androgen replacement in elderly men with sarcopenia and low T has been reported to increase muscle strength in several, but not all, clinical trials^12,13^. T therapy in elderly men remains controversial because of the potential side effects on the cardiovascular system and prostate^14^. Given this background, the positive impact of T stimulating physical activity and the underlying mechanisms of action represent an additional consideration for T replacement in elderly, mobility-impaired, frail or sarcopenic elderly men.

Animal models represent an indispensable tool to understand how T influences locomotor behavior. Both orchidectomy (ORX) and knock out (KO) of the androgen receptor (AR) in mice replicate several behavioral features of sex steroid deficiency in humans^15^. Wheel running is a rewarding behavior observed even in wild rodents and other animals^16^. In mice, we and others have reported an almost complete abrogation of wheel running activity after ORX and in ARKO^17,18^. However, a neuromuscular dysfunction resulting from the loss of androgenic stimulation could alternately explain the low wheel running phenotype in ORX and ARKO mice. The anabolic effects of androgens on muscle mass are well-documented in men, although in mice limb muscle appear less androgen-sensitive^19^. Thus, the contribution of the central (motivation) over the peripheral (neuromuscular) actions of androgens on activity needs to be elucidated.

Another open issue regarding the actions of T on physical activity behavior is the role for free T. By using a transgenic (Tg) mouse model expressing human SHBG^20^, we recently demonstrated that T is “trapped” in the circulation by SHBG and cannot enter into target tissues to elicit its physiological functions^21^. The androgen deficiency phenotype in SHBG-Tg mice was however attenuated compared to ORX^21^. Therefore, the SHBG mouse model represents a unique approach to study the impact of free T whilst replicating better than other “complete” androgen deficiency models the moderate drop in the T levels as they occur in ageing men.

T can directly, or indirectly, following conversion to dihydrotestosterone (DHT), act via the androgen receptor (AR). T, but not DHT, can also be converted into 17β-estradiol (E2) by the aromatase enzyme and activate estrogen receptors (ERs). Aromatization is an important determinant of many of the actions of T^22^. In male mice, the circulating levels of E2 are negligible^21^ and local aromatization of T within different tissues including the brain is believed to constitute a key mechanism of estrogenic action. For instance, T acts predominantly as a prohormone for E2 in the programming of male-typical and territorial behavior in mice, although execution of this behavior relies on the AR^23^. It remains debated whether this dual androgenic and estrogenic action of T also applies to wheel running. A couple of studies in mice have indicated such a dual mode of action of T on promoting wheel running^6,7^, although this hypothesis could not be proven by other authors^24^.

Although it is clear that sex steroids are important neuroendocrine regulators of physical activity behavior, the underlying mechanisms remain poorly understood. The brain dopamine (DA) system is suggested as the final common pathway for a complex network of neuromediators contributing to the modulation of physical activity. In particular, the activation of DA receptor 1(DR1)- and DR2-neurons in the striatum exerts a selective control over locomotion^25^. Despite evidence indicating a role for T in DA synthesis, release and metabolism, results are mostly inconsistent between studies^26^. Therefore, further studies are warranted to better understand the neuroendocrine interaction between androgens and the central DA system.

The aim of this study was to examine how T regulates voluntary physical activity. In particular, our goal was to answer the four following questions: (1) does T-induced stimulation of activity depend in part on its aromatization into E2? (2) is the free fraction of T mediating the actions on activity? (3) to what extent are the effects of androgens exerted through central mechanisms (motivation) independent from peripheral (neuromuscular) actions? and (4) are the brain DA pathways implicated in the T regulation of activity? For this purpose, we explored wheel running, home-cage activity and the neuromuscular function in two mouse models of androgen deficiency (ORX and ARKO) given placebo, T or dihydrotestosterone (DHT) replacement, as well as in SHBG-Tg mice. The implication of the DA system in the T-mediated regulation of physical activity was determined *in vivo* by monoamine profiling, gene expression studies and pharmacological challenging with dopaminergic drugs.

## Results

### Testosterone is more effective than dihydrotestosterone in restoring wheel running in ORX mice

**Figure 1:**
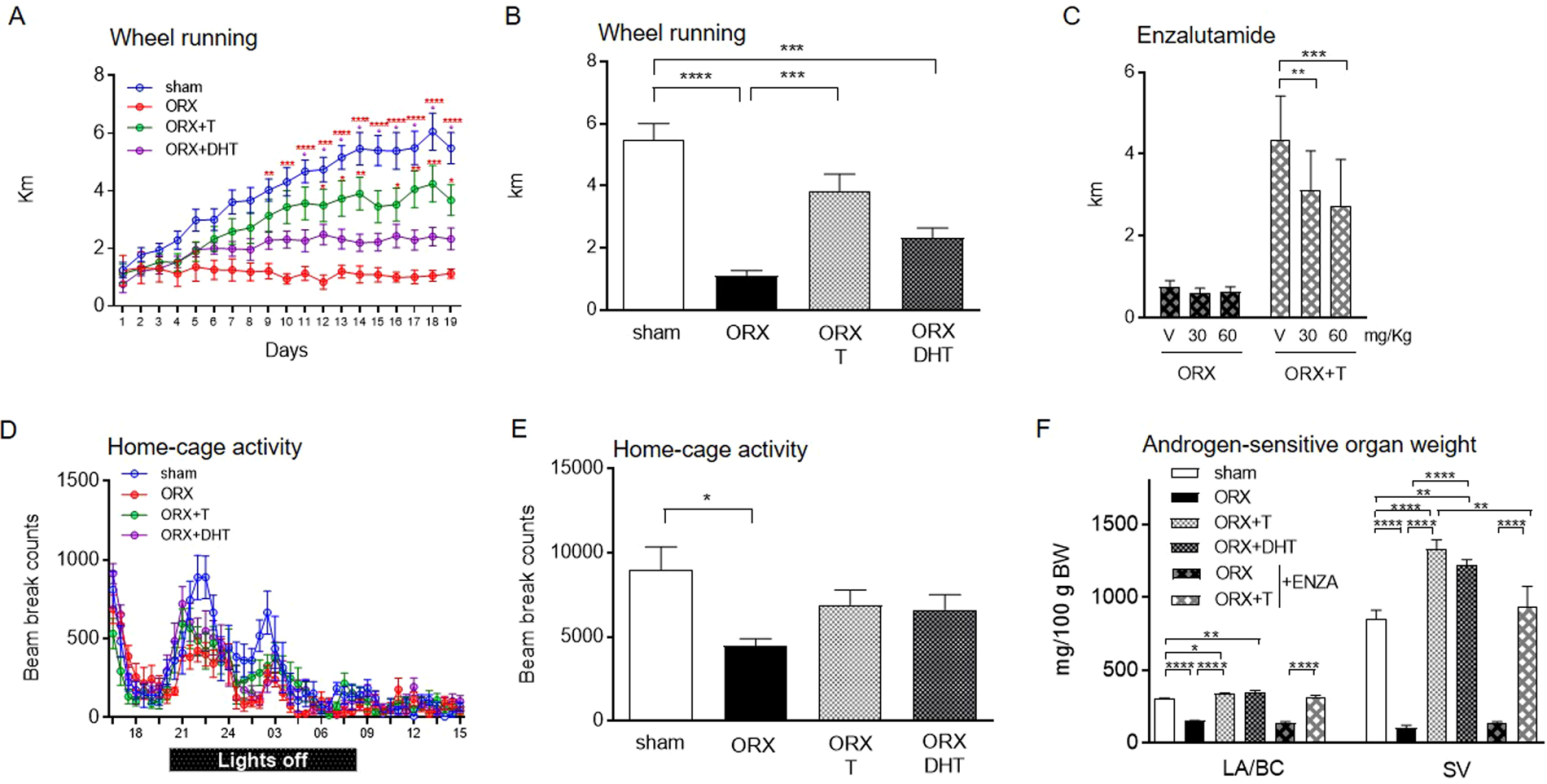
Effects of androgens on physical activity in C57BL/6J male mice. Time course of daily wheel running activity over a period of 19 days (**A**) and average distance run per day after two weeks of training (**B**). Note that the color of the stars in A represents the specific comparisons. Average distance run per day after two weeks of training during exposure to vehicle and treatment with increasing doses of enzalutamide (**C**). Ambulatory movement beam breaks over 23 h (**D**) and total cumulative ambulatory activity during the night (**E**). (**F**) Seminal vesicle and LA/BC weight following ORX and replacement with T or DHT. Data in panels A and C were analyzed using two-way repeated measures ANOVA and those in panels B and E–F were analyzed by one-way ANOVA. Data are presented as mean ± SEM. In (**A,B**) and (**F**), n = 5–10 animals per group; in (**C**), n = 6 animals per group; in (**D**,**E**), n = 10–11 animals per group. Statistical significance levels: ^*, **, ***, ****^P < 0.05, 0.01, 0.001 and 0.0001. ENZA enzalutamide, LA/BC levator ani/bulbocavernosus, SV seminal vesicles.

When C57BL/6J sham mice were given free access to a running wheel, they steadily increased their activity each day until they reached a plateau at 2 weeks (Fig. 1A). As expected, orchidectomy (ORX) completely abrogated the progressive increase in the daily running distance [androgens: F(3,29) = 9.252, P = 0.0002; time: F(18,522) = 32.11, P < 0.0001; interaction: F(54,522) = 7.345, P < 0.0001; Fig. 1A]. Supplementation with T induced an almost 250% increase in the activity levels during the plateau phase compared to ORX (ORX: 1.09 ± 0.18 km; ORX + T: 3.8 ± 0.57 km; P < 0.001; Fig. 1B), restoring wheel running almost to sham levels (P > 0.05 *vs*. sham). DHT also augmented the running distance in ORX animals (Fig. 1A and B) but to a lesser extent, not reaching statistical significance. Indeed, Bonferroni post hoc analysis revealed that DHT-treated mice significantly ran less compared to sham animals (sham: 5.49 ± 0.51 km; ORX + DHT: 2.31 ± 0.31 km; P < 0.001; Fig. 1B). Still, treatment with the AR antagonist enzalutamide diminished wheel running activity by 40% in ORX + T but not ORX mice [T: F(1,10) = 7.140, P < 0.05; enzalutamide: F(2,20) = 7.532, P < 0.01; interaction: F(2,20) = 5.307, P = 0.0142; Fig. 1C].

Similar but attenuated effects for ORX and hormone replacement were observed in home-cage activity, which was reduced by ORX and restored by T as well as DHT (Fig. 1D and E). When evaluating the peak of ambulatory activity during dark hours, sham animals were found to be more active than ORX (sham: 9005 ± 1355 beam counts; ORX: 4457 ± 455 beam counts; P < 0.05; Fig. 1E). No differences were observed between sham and ORX + T- or DHT-treated mice (Fig. 1E).

The effectiveness of ORX, hormone replacement and treatment with enzalutamide was demonstrated by determining the weight of the androgen-sensitive seminal vesicle and levator ani/bulbocavernosus (LA/BC) muscle complex (Fig. 1F).

### SHBG inhibits the stimulatory effects of testosterone on wheel running activity

**Figure 2:**
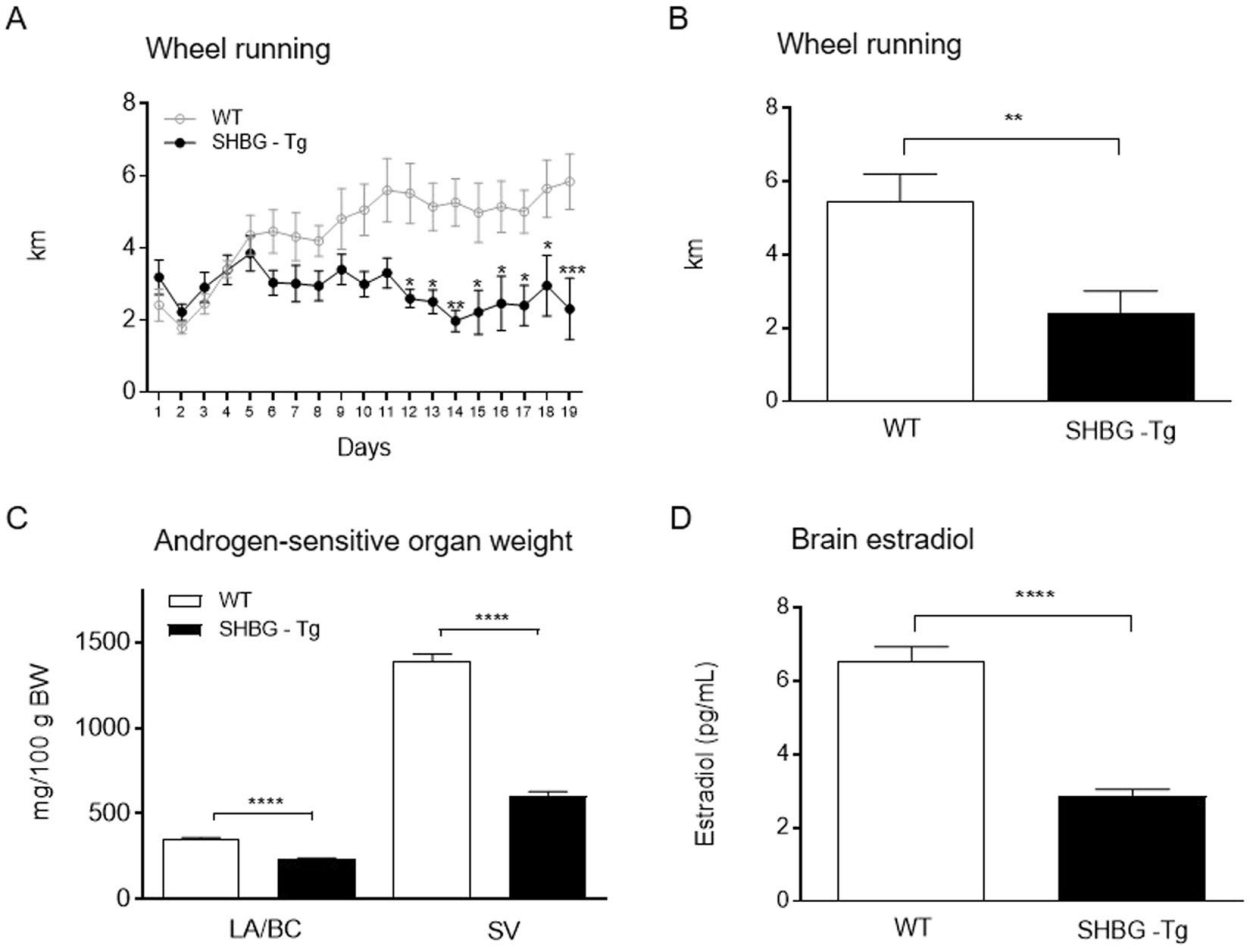
SHBG inhibits the stimulatory effects of T on wheel running activity. Time course of daily wheel running activity over a period of 19 days (**A**), average distance run per day after two weeks of training (**B**), seminal vesicle and LA/BC weight (**C**) and concentration of E2 in brain homogenates (**D**) in SHBG-Tg and WT mice, both treated with T following ORX. Data in panel A were analyzed using two-way repeated measures ANOVA and those in panels B,D were analyzed by Student t-test. All data are mean ± SEM, 6 animals per group. Statistical significance levels: ^*, **, ***, ****^P < 0.05, 0.01, 0.001 and 0.0001. LA/BC levator ani/bulbocavernosus, SV seminal vesicles.

To determine the contribution of free T to the actions of T we assessed wheel running after ORX and T replacement in both sex hormone-binding globulin (SHBG)-Tg and wild type (WT) littermate controls. SHBG-Tg mice did not display the progressive increase in the daily running distance as observed in their WT littermates [genotype: F(1,10) = 7.806, P = 0.0190; time: F(18,180) = 4.325, P < 0.0001; interaction: F(18,180) = 5.451, P < 0.0001; Fig. 2A]. Expression of SHBG reduced the activity levels by 56% during the plateau phase (P ≤ 0.01; Fig. 2B). As previously described^21^, SHBG-Tg mice showed a mild hypogonadism, confirmed by a 34% and a 56% decrease in the weight LA/BC muscles and seminal vesicles, respectively (P < 0.0001 *vs*. WT; Fig. 2C). In a similar pattern, we observed a 57% reduction in the concentration of E2 in the brain of SHBG-Tg mice (P < 0.0001 *vs*. WT; Fig. 2D). No differences in brain aromatase expression were found between genotypes (data not shown). Also in SHBG-Tg mice, E2 levels in the brain were strongly associated (R^2^ = 0.76, P = 0.02) with the average distance run per day (Supplementary Fig. 1). A significant correlation between the two parameters was also observed for all animals but not for WT group alone (Supplementary Fig. 1).

### Testosterone stimulation of wheel running is independent of its action on muscle

**Figure 3:**
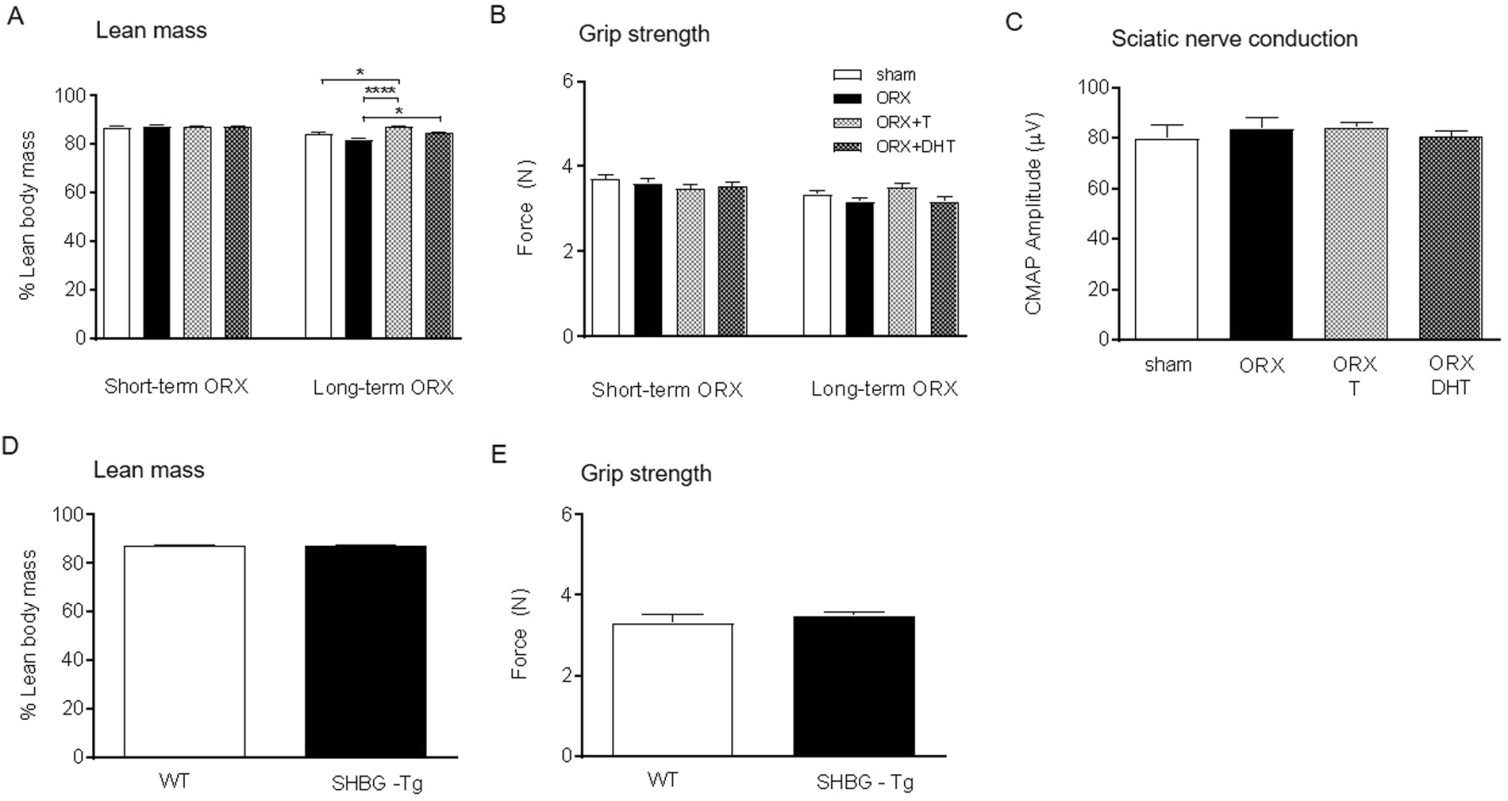
Testosterone stimulation of wheel running is independent of its peripheral action. Percentage of lean mass by DEXA (**A,D**) and total-limb maximal grip strength (**B**,**E**) in C57BL/6J (**A,B**) and SHBG-Tg and WT mice (**D,E**). Mean sciatic CMAP amplitudes after ORX and replacement with T or DHT in C57BL/6 J mice (**C**). Data in panel A–C were analyzed using one-way ANOVA and those in panels D,E were analyzed by Student t-test. Data are presented as mean ± SEM. In A–C, n = 5–9 animals per group; in (**D**,**E**), n = 6 animals per group. Statistical significance levels: ^*, ****^P < 0.05 and 0.0001. CMAP compound muscle action potential.

We next tested if the stimulatory actions of T on wheel running activity in male mice were associated with an improvement of their physical ability. At the end of the wheel running period (short-term ORX), neither ORX nor hormone replacement in C57BL/6J male mice altered the percentage of lean tissue mass by DXA (Fig. 3A) or grip strength (Fig. 3B). Also at this stage, androgens had no effect on peripheral nerve function, as determined *in vivo* by analyzing the compound muscle action potentials (CMAPs) amplitude elicited by the stimulation of the sciatic nerve (Fig. 3C). When animals were kept two months post-ORX (long-term ORX), we observed an effect of T, and to a lesser extent of DHT, increasing lean mass (P < 0.0001 and P < 0.05 *vs*. ORX, respectively; Fig. 3A), although there was only a tendency to a better performance in the grip strength test (P = 0.076; Fig. 3B). Similarly, the values of lean tissue mass (Fig. 3D) and grip strength (Fig. 3E) were indistinguishable between short-term ORX + T-treated SHBG-Tg and WT mice, consistent with previous reports^21^.

To prove that myogenic AR was not driving the effects of T on wheel running, we compared wheel running activity of male WT and conditional satellite-cell lineage ARKO (satARKO) mice, both treated with T following ORX. The disruption of AR in satARKO mice is restricted to skeletal muscle^27^. In consistence with our previous results^27^, satARKO mice displayed normal hindlimb muscle weight but a 50% reduction in the mass of LA/BC (P < 0.0001 *vs*. WT; Supplementary Fig. 2A). Wheel running patterns were comparable between genotypes, thus demonstrating that myogenic AR is dispensable for wheel running in male mice (Supplementary Fig. 2B).

### Testosterone increases wheel running activity even in the absence of AR

**Figure 4:**
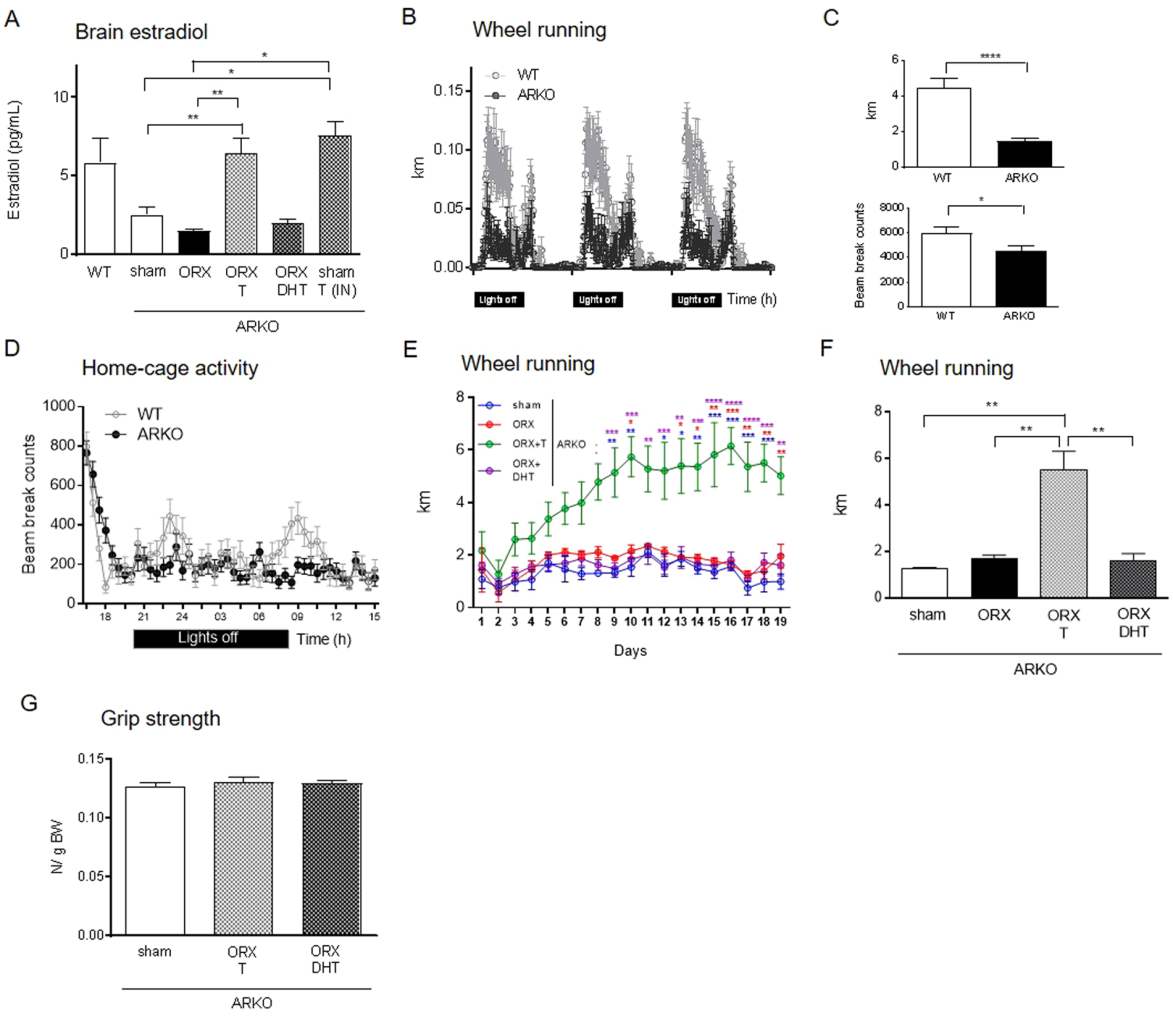
Testosterone restores wheel running activity even in the absence of AR-signaling. (**A**) Concentration of E2 in brain homogenates in WT and ARKO mice after both ORX and hormone replacement or intranasal treatment with T. Circadian patterns of wheel running (**B**) and average distance run per day (C, upper panel) in trained ARKO and WT mice. Ambulatory movement beam breaks over 23 h (**D**) and total cumulative ambulatory activity during the night (**C**, lower panel). Time course of daily wheel running activity over a period of 19 days (**E**) and average distance run per day after two weeks of training (**F**) in ARKO mice after ORX and hormone replacement. Note that the color of the stars in (**E**) represents the specific comparisons. Total-limb maximal grip strength (**G**) in sham, ARKO + T and ARKO + DHT mice. Data in panel A, F and G were analyzed using one-way ANOVA and those in panels C and E were analyzed by Student t-test and two-way repeated measures ANOVA, respectively. Data are presented as mean ± SEM. In (**A**) n = 3–14 animals per group; in (**B** and **C**) (upper panel), n = 18 animals per group; in C (lower panel) and D, n = 17–21 animals per group; in (**E** and **F**), n = 3–6 animals per group; in G n = 5–8 animals per group. Statistical significance levels: ^*, **, ***, ****^P < 0.05, 0.01, 0.001 and 0.0001.

Our results in ORX mice supplemented with T and DHT as well as in ORX + T-treated mice receiving enzalutamide suggested a dual role (androgenic and estrogenic) for T in the regulation of wheel running. To confirm the ability of T to stimulate activity in an AR-independent manner, we performed ORX and hormone replacement studies in male mice lacking the AR. We first ruled out the presence of other behavioral alterations in androgen receptor KO (ARKO) mice besides locomotion. No differences between genotypes were observed when evaluating standard measures of anxiety in the elevated plus maze (Supplementary Fig. 3A) nor in the test of spatial memory Morris Water maze (Supplementary Fig. 3B and C). We also confirmed the ability of ARKO mice to aromatize T. Following an acute administration of intranasal T, there was a 2.5 fold increase in brain E2 levels of ARKO mice (sham: 2.88 ± 0.72 pg/mL; T: 7.57 ± 0.89 pg/mL; P < 0.05; Fig. 4A), which otherwise were low compared to WT.

Intact ARKO mice showed a 70% reduction in wheel running (WT: 4.44 ± 0.52 km; ARKO: 1.47 ± 0.16 km; P < 0.001; Fig. 4B and C) and a 24% decrease in home-cage activity (WT: 5930 ± 550 beam counts; ARKO: 4499 ± 389 beam counts; P < 0.05; Fig. 4C and D). As expected, the low wheel running phenotype in ARKO mice was maintained after supplementation with DHT (Fig. 4E and F). In contrast, T replacement resulted in a rapid and dramatic enhancement of wheel running activity [treatment: F(3,13) = 8.763, P = 0.0019; time: F(18,234) = 8.497, P < 0.0001; interaction: F(54,234) = 4.124, P < 0.0001; Fig. 4E], reaching a 225% increase during the plateau phase compared to ORX (P < 0.01 *vs*. ORX; Fig. 4F). The effectiveness of T supplementation was confirmed by higher E2 levels in brain homogenates (Fig. 4A).

The low activity phenotype in ARKO animals was not associated with any alteration in grip strength, rotarod test performance, peripheral nerve function or motor neuron counts in the spinal cord (Supplementary Fig. 4). Indeed, despite the dramatic increase in wheel running in ORX + T-treated ARKO mice, these animals showed an indistinguishable grip strength compared to sham and ORX + DHT groups (Fig. 4G).

### Testosterone attenuates the in vivo sensitivity to amphetamine

Our results show that T promotes physical activity in males independently of its peripheral action. Thus, the next step was to test the “central hypothesis”, namely that T is acting on brain circuitries responsible for motor movement, including the striatal monoaminergic systems.

**Figure 5:**
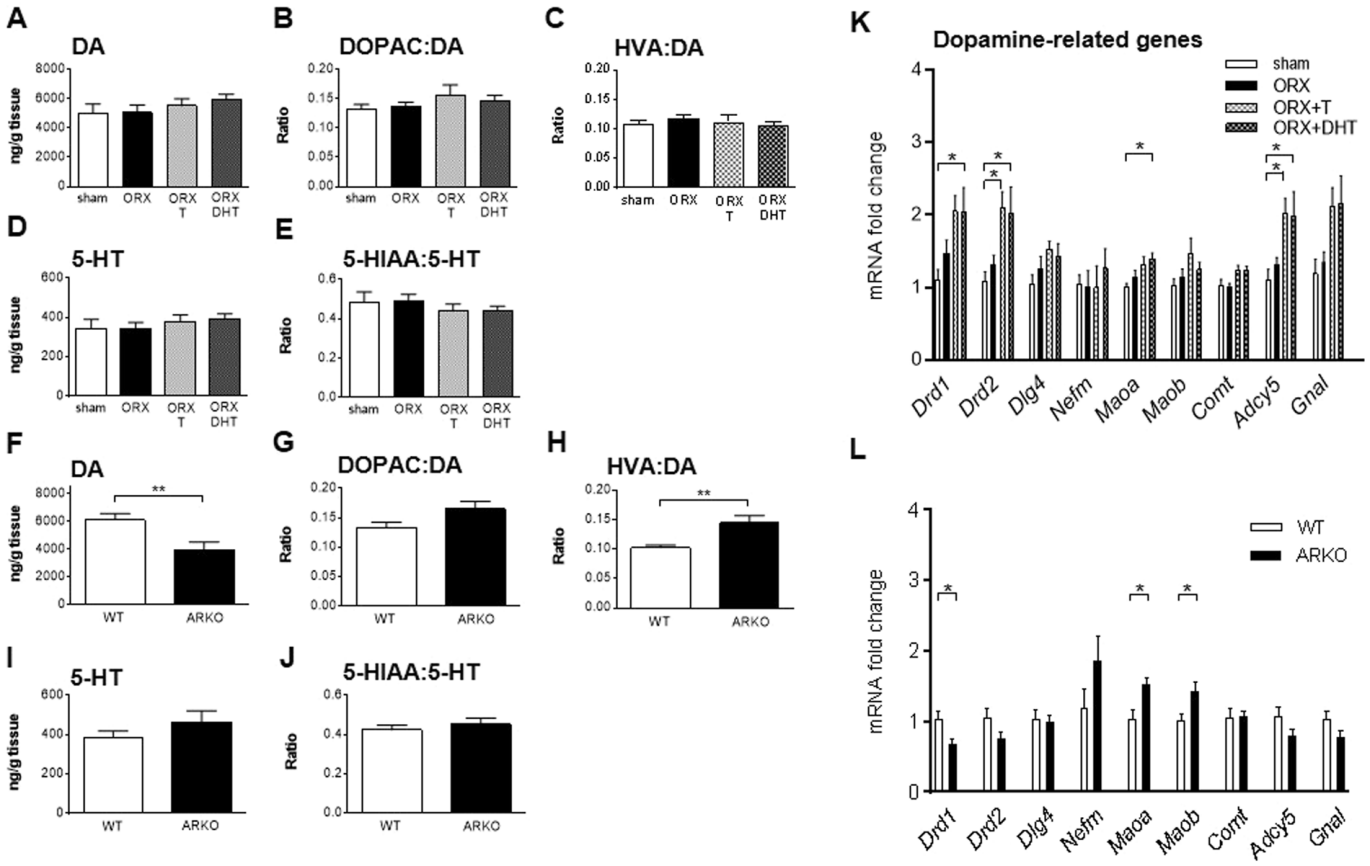
Effects of androgens on the striatal monoaminergic system. DA (**A**,**F**), DOPAC to DA ratio (**B**,**G**), HVA to DA ratio (**C**,**H**), 5-HT (**D**,**I**) and 5-HIAA to 5-HT ratio (**E**,**J**) were measured by HPLC in the dorsal striatum of C57BL/6J (**A**–**E**) and ARKO and WT mice (**F**–**J**). Quantitative real-time PCR analysis of striatal mRNA levels of dopamine receptor 1 (*Drd1*) and 2 (*Drd2*), postsynaptic density protein 95 (*Dlg4*), neurofilament, medium polypeptide (*Nefm*), monoamine oxidase A (*Maoa*) and B (*Maob*), catechol-O-methyltransferase (*Comt*), adenylate cyclase (*Adcy5*) and olfactory isoform of the stimulatory GTP-binding protein alpha subunit (*Gnal*) of C57BL/6J (**K**) and ARKO and WT mice (**L**). Data in panels A–E and K were analyzed using one-way ANOVA and those in panels F–J and L were analyzed by Student t-test. Data are presented as mean ± SEM. In (**A**–**E**), n = 7–9 animals per group; in F–J, n = 10 animals per group; in K, n = 6–9 animals per group; in L, n = 7 animals per group. Statistical significance levels: ^*, **^P < 0.05 and 0.01. 5-HT serotonin, 5-HIAA hydroxyindolacetic acid, DA dopamine, DOPAC 3,4-dihydroxyphenylacetic acid, HVA homovanillic acid.

HPLC analysis revealed that neither ORX nor hormone replacement in C57BL/6J mice altered the striatal tissue content of dopamine (DA), 3,4-dihydroxyphenylacetic acid (DOPAC), homovanillic acid (HVA), serotonin (5-HT), 5-hydroxyindolacetic acid (5-HIAA), or the metabolite/monoamine ratios (Fig. 5A–E). However, ARKO mice displayed lower DA striatal levels compared to WT littermates (WT: 6119 ± 451 ng/g tissue; ARKO: 3944 ± 580 ng/g tissue; P < 0.01; Fig. 5F), a decrease associated to an enhanced DA metabolism, as indicated by an increased HVA/DA ratio (Fig. 5H). Also in ARKO mice, the content of 5-HT and its metabolites remained unaltered (Fig. 5I–J). Regardless of the experimental group, the concentrations of noradrenaline (NAD) in the dorsal striatum were often below the limit of detection of the HPLC method and therefore are not shown.

As previously described^28^, ORX had no effect on the transcript levels of DA-related genes in the striatum, as assessed by qPCR analysis (Fig. 5K). Still, hormone replacement increased the expression of several DA-related genes, reaching statistical significance for *Drd2* and adenylyl cyclase type 5 (*Adcy5*) in ORX + T, and for both *Drd1* and *Drd2*, the DA metabolic enzyme monoamine oxidase A (*Maoa*) and *Adcy5* in ORX + DHT (Fig. 5K). Regarding ARKO mice, the deletion of AR reduced the mRNA levels of DA receptors, although statistical significance was only reached for *Drd1*, and increased the expression of the metabolic enzymes *Maoa* and *Maob* (Fig. 5L).

We also evaluated if T modulated DA-sensitive behaviors *in vivo*. To this end, mice were challenged with an injection of amphetamine, which increases DA concentrations in the synapse and induces a hyperlocomotion response.

**Figure 6:**
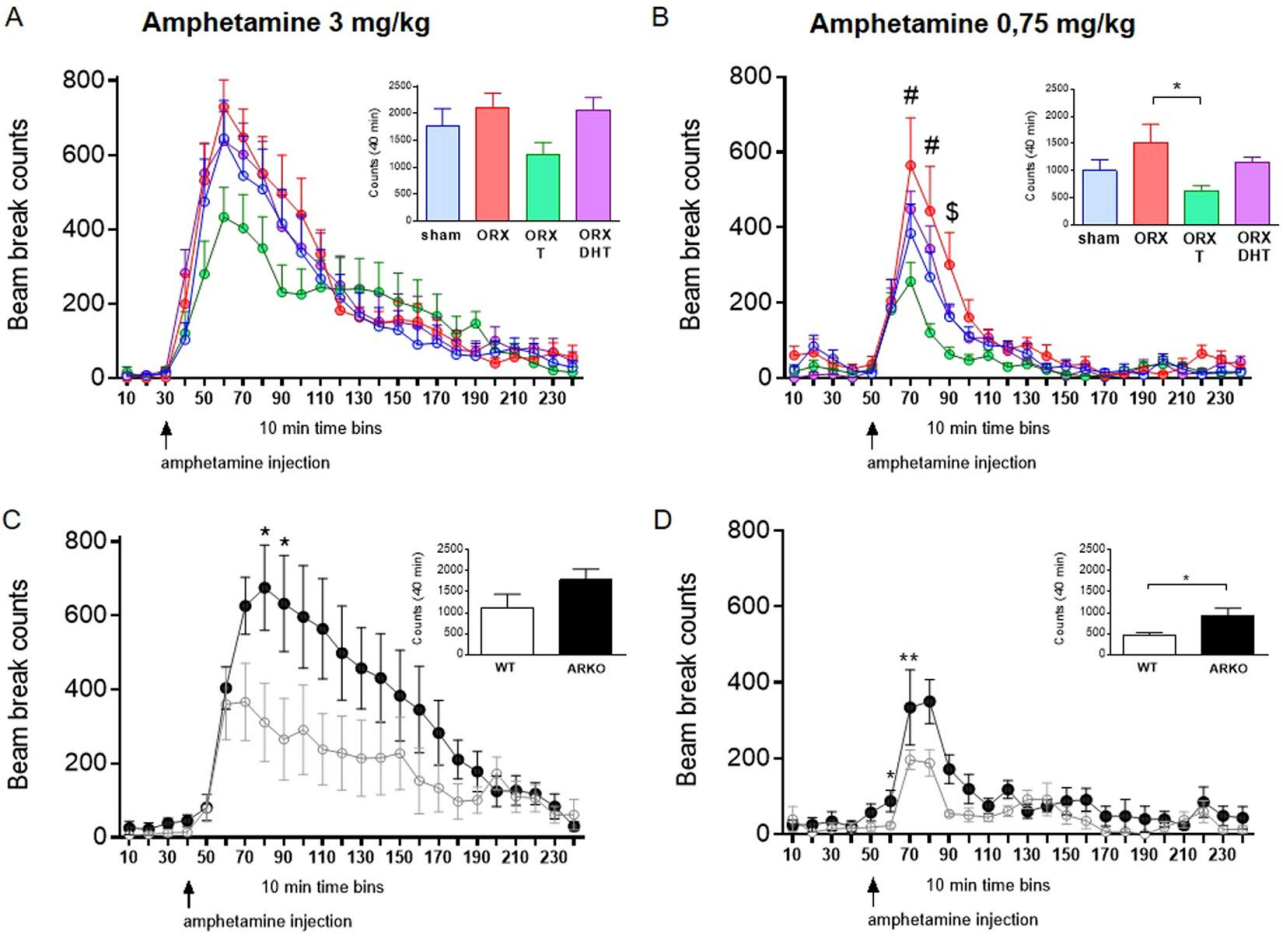
Androgen deficiency aggravates and treatment with T attenuates the locomotion response to amphetamine. Time course of locomotor-induced behavior after i.p. injection with 3 (**A**,**C**) or 0.75 mg/kg (**B**,**D**) amphetamine in C57BL/6 J (**A**,**B**) and ARKO and WT mice (**C**,**D**). The inset graphs show the cumulative beam breaks during 40 min. Locomotor activity evolution was analyzed using two-way repeated measures ANOVA. Cumulative activities were analyzed using one-way ANOVA or Student t-test. Data are presented as mean ± SEM. In (**A**,**B**), n = 10–11 animals per group; in (**C**,**D**), n = 7–8 animals per group. Statistical significance levels: ^*, **^P < 0.05 and 0.01 *vs*. WT. ^#^P < 0.05 *vs*. sham and ORX T. ^$^P < 0.05 *vs*. ORX T.

The evolution in the beam break counts after administration of amphetamine at a dose of 3 mg/kg was similar between sham and ORX + placebo- or DHT-treated mice (Fig. 6A). However, ORX + T mice displayed a blunted response compared to the rest of the groups [interaction: F(69,851) = 1.968, P < 0,05; Fig. 6A]. Two-way repeated-measures ANOVA revealed a significant main effect of androgens, time as well as their interaction on the amount of locomotor activity following injections of 0.75 mg/kg amphetamine [androgens: F(3,37) = 4.216, p = 0.0116; time: F(23,851) = 46.24, p < 0.0001; interaction: F(69,851) = 1.968, p < 0.0001]. Post hoc analysis revealed that ORX mice displayed a more pronounced locomotion response *vs*. sham animals at both 20 and 30 min post-injection (P < 0.01 and 0,05, respectively; Fig. 6B), an effect totally prevented by T (P < 0.0001 *vs*. ORX; Fig. 6B) but not DHT. As shown in the inset of Fig. 6B, the 40 min cumulative locomotor activity post-injection was significantly decreased in ORX + T- compared to ORX + placebo-treated mice. In a similar pattern, ARKO mice displayed an enhanced locomotion response to amphetamine at both 0.75 and 3 mg/kg (Fig. 6C and D).

Finally, we tested if T-mediated modulation of the locomotion response to amphetamine was driven by non-genomic rapid mechanisms, as suggested for other T-dependent behaviors^29^. Intranasal T increased T levels in both serum and brain after a few minutes (Supplementary Fig. 5A). However, short-term exposure to intranasal T did not alter the hyperlocomotion response to 3 mg/kg amphetamine in ORX mice (Supplementary Fig. 5B).

### Dopamine receptor antagonism selectively reduces the effects of testosterone on wheel running

**Figure 7:**
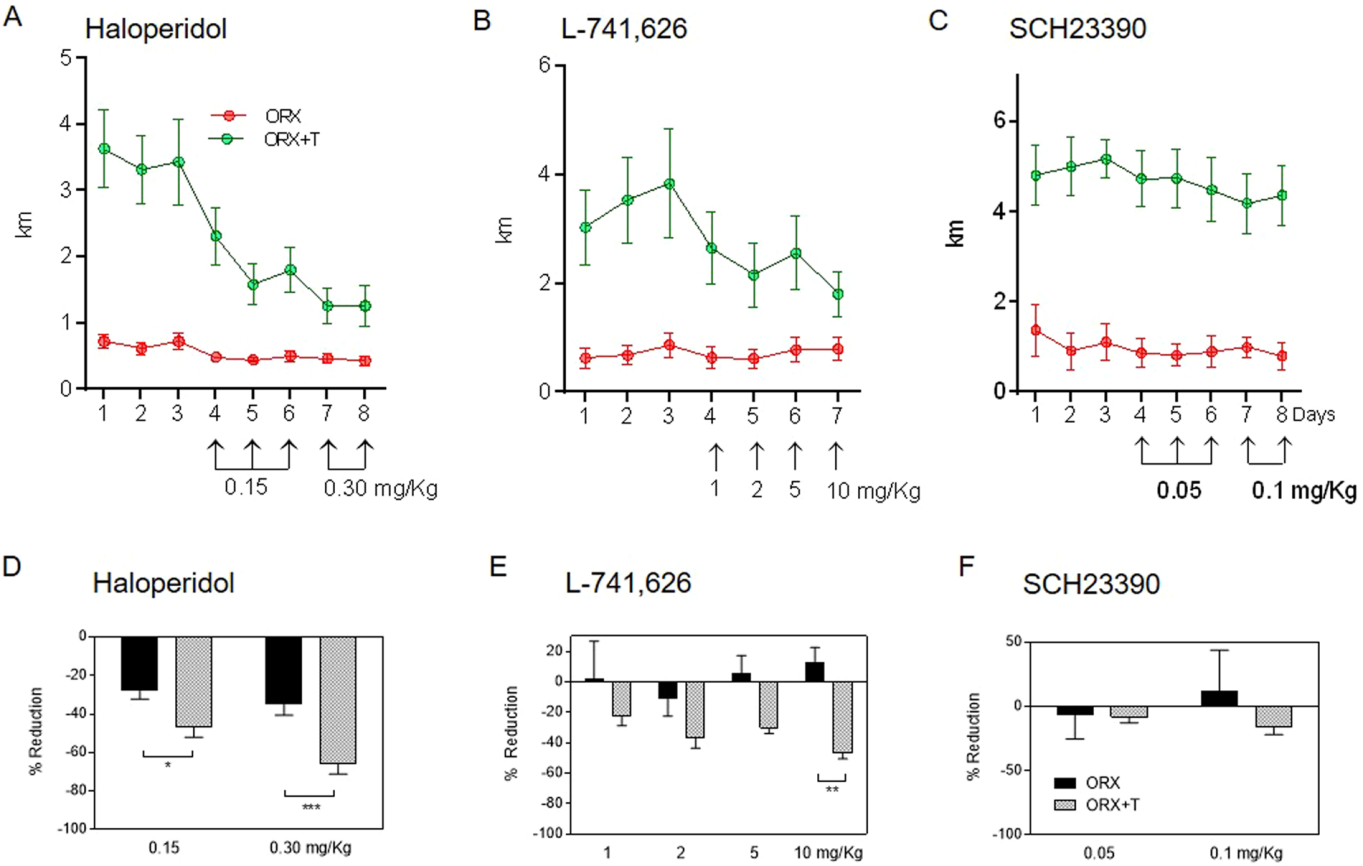
DR2 antagonism inhibits T-induced stimulation of wheel running. Running-wheel activity during baseline (first three days) and after treatment with increasing doses of haloperidol (**A**), L-741,626 (**B**) and SCH23390 (**C**). Percentage of reduction from baseline after exposure to haloperidol (**D**), L-741,626 (**E**) and SCH23390 (**F**). Data in panels D–F were analyzed using two-way repeated measures ANOVA. Data are presented as mean ± SEM. In (**A** and **D**), n = 8–11 animals per group; in B and E, n = 6 animals per group; in (**C** and **F**), n = 4–6 animals per group. Statistical significance levels: ^*, **, ***^P < 0.05, 0.01 and 0.001.

To confirm the role of DA on the actions of T on wheel running, we challenged both ORX + placebo- and ORX + T-treated mice with DA antagonists. The addition of the DR2 antagonist haloperidol in the drinking water elicited a dose-dependent suppression of wheel running (Fig. 7A and D). Two-way repeated-measures ANOVA revealed a significant main effect of T, haloperidol dose as well as their interaction on the relative drop of wheel running [T: F(1,17) = 12.4, p = 0.0026; dose: F(1,17) = 19.76, P < 0.001; interaction: F(1,17) = 4.776, P < 0.05, Fig. 7D]. The decrease of activity in ORX + T-treated mice after two increasing doses of haloperidol was enhanced by 68 and 92%, respectively, compared to ORX + placebo group (Fig. 7D). Similarly, i.p. injections with increasing doses of the DR2 antagonist L-741,626 resulted in an inhibition of wheel running but only in ORX + T mice [T: F(1,10) = 15.18, P < 0.01; dose: F(3,30) = 0.59, P > 0.05; interaction: F(3,30) = 1.104, P > 0.05, Fig. 7B and E].

Finally, treatment with the DR1 antagonist SCH23390 exerted only a marginal reduction in the distance run, regardless of whether mice were receiving T replacement or placebo (Fig. 7C and F).

## Discussion

The etiology of physical activity in humans has been suggested to be multi-factorial, involving complex interactions between individual, social, and environmental elements^30^. Of particular interest is the role of potential biological factors determining the willingness/ motivation to engage in exercise. The effects of castration abrogating wheel running in rodents were already reported decades ago^24^. However, little progress has been made since then in our knowledge of how T regulates physical activity. The results of the present study clearly show the central nature of T actions on wheel running, whilst confirming that the contribution of muscle AR is marginal. Also, our findings point towards a role for T as an estrogenic prohormone in the regulation of activity. Furthermore, we show for the first time the regulation of wheel running via the free fraction of T, confirming the free hormone hypothesis for T. Finally, our results demonstrate that T-induced stimulation of wheel running is dependent on the DA system.

This study is the first systematic approach to address activity behavior by both wheel running and home-cage activity and this as well in ORX as ARKO mice. The two locomotion parameters have been often considered as interchangeable in rodents. However, evidence suggests that wheel running is not solely a motor response but is rather an incentive-motivated behavior. For instance, rats will press a lever to gain access to a running wheel^31^ and will develop preference for environments previously associated with wheel running^32^. Indeed, wheel running has been used as an animal model to study the mechanisms underlying the “runner’s high” in humans^32^. The motivational nature might account for the differences in the impact of androgen deficiency on wheel running and home cage activity. Both ORX and ARKO mice showed a reduction in the two locomotion parameters but the decrease was far more pronounced in wheel running. In contrast to previous studies^6,7^, the post-ORX recovery period in this study was minimal (48 h) and the early pre-plateau phases were also included in the analysis. We hereby were able to show that several wheel running parameters were already altered within the first week after castration. This rapid and dramatic action of ORX places physical activity as a highly androgen-sensitive parameter in male mice, similar even to classical androgen-responsive reproductive organs.

Compared to total T concentrations, free levels of T are a better predictor of deficiency–related symptoms in elderly men with late-onset hypogonadism^10^. The impact of the free fraction of T on wheel running was investigated by using SHBG-Tg mice^20^. This approach also allowed us to compare the results of ORX and ARKO mice to a less “complete” androgen deficiency model. When provided with an equal amount of T, SHBG-Tg mice display a decrease in the free levels of T due to SHBG binding^21^. Under these circumstances, SHBG-Tg mice showed a low wheel running phenotype associated to a reduced systemic androgen bioactivity, as evidenced by seminal vesicle weight. In addition, their brain levels of E2 were diminished despite a similar expression of brain aromatase *vs*. WT, thereby indicating a reduced availability of substrate T to enter the brain from the circulation and become locally aromatized into E2, which is in line with our previous observations^21^. The decline of total T in ageing is only moderate whereas the rise in SHBG concentrations and decline in free T are more pronounced^9^. Therefore, SHBG-Tg mice may be a more suitable animal model to study late-onset hypogonadism than ORX. Despite the obvious limitations of comparing data across different models, it is remarkable that SHBG-Tg mice still run slightly more than ORX mice. Therefore, the T-induced boosting of wheel running in male mice might be fine-tuned by a concentration-response pattern. In accordance, we observed a significant association between wheel running and E2 levels in the brain, particularly at low concentrations (SHBG-Tg mice). However, further studies using different T concentrations are warranted to corroborate this hypothesis.

Many of the actions of T in male behavior are mediated by ERs^22^. In male mice, the circulating levels of E2 are negligible^21^ while aromatase is highly expressed in the brain, suggesting a role for the autocrine and paracrine actions of estrogens^33^. Regarding wheel running, the extent to which AR and ERs contribute to the T-induced stimulation of activity remains unclear. In ORX rats, wheel running was unaffected by the non-aromatizable DHT while it dramatically increased after treatment with E2^24^. Still, treating male rats with systemic E2 is a rather poor physiological approach since their serum E2 concentrations are extremely low^34^. More recent studies in mice^6,7^ showed a moderate but consistent effect of DHT in rescuing wheel running after castration. In accordance, our experiments with both DHT and enzalutamide indicated that AR-signaling stimulates wheel running in male mice, yet still not sufficient to explain the full action of T. DHT can be metabolized *in vivo* to 3α-diol and 3β-diol, which display a weak AR binding activity and act through the gamma-aminobutyric acid (GABA) type A and/or ERs to initiate biological responses^35,36^. We proved here that the weak actions of DHT on wheel running were mediated through AR mechanisms since these were no longer observed in ARKO mice. We next challenged ARKO mice with T to determine the implication of AR-independent mechanisms. This approach was chosen over systemic treatment with E2 since circulating E2 is unphysiological in mice, while T treatment in ARKO mice better replicates the suggested local nature of the estrogenic action in male mice^21,33^. Even in the absence of AR, treatment with T increased wheel running in mice, an effect that was associated with an up-regulation of brain E2 levels. Although we cannot fully exclude the possibility that T actions on global ARKO mice imply other mechanisms outside ERs pathways, this seems unlikely since the non-aromatizable androgen DHT did not recapitulate T effects. Therefore, our results point to a full action of T on wheel running dependent on prior aromatization into E2.

The modulation of wheel running in male mice by androgens could be reflective of changes in their overall physical ability. Our results did not indicate an impaired neuromuscular function in any of the 3 mouse models of androgen deficiency. Still, we cannot rule out potential alterations in the intrinsic contractile properties of skeletal muscle since we did not include a “pure” readout of muscle strength. Both ORX^37^ and AR deletion^38^ result in a mild reduction in the mass as well as the *in vitro* contractility of hindlimb muscles. However, this is not translated into an alteration of the *in vivo* endurance capacity^17,39^. Thus, it seems unlikely that the beneficial effects of androgens on muscle intrinsic properties could explain the stimulatory actions of T on wheel running activity. Supporting our view, satARKO and control mice displayed comparable levels of wheel running, demonstrating that myogenic AR is dispensable for wheel running in male mice.

We next tested the “central hypothesis”, namely that T mainly was acting on brain circuitries to stimulate wheel running. Voluntary exercise is likely regulated by a complex network of neuromediators but the DA system has been suggested as the final common pathway. Both optogenetics^25^ and designer receptors exclusively activated by designer drugs (DREADD)^40^ studies corroborate the activation of DR1- and DR2-neurons in the striatum as a key determinant for locomotion. Castration and T replacement in male rodents have been associated to changes in DA synthesis, transport, release, metabolization and/or signaling, despite the direction of the changes varies depending on the brain region and time post-castration assessed^26^. In humans, PET studies suggest the existence of sex differences in DA function^41^. We chose to explore the dorsal striatum based on previous findings showing that the alterations in the DA system associated to wheel running hyperactivity are more pronounced in this region than in the nucleus accumbens^42^. Accumulated data reveal a previously neglected role for the nigrostriatal pathway facilitating motivational behaviors^43^. Here, we show a substantial loss of DA in the striatum of ARKO mice, associated with an increased metabolism ratio. Also in ARKO, there was a reduction in the striatal expression of DA receptors while the mRNA levels of the metabolic enzymes *Maoa* and *Maob* were up-regulated. Therefore, a diminished DA tone could be the neural basis underpinning the low locomotion phenotype of ARKO mice. Unexpectedly, ORX *per se* however had no effects either on DA concentrations or on DA-related genes in the striatum, despite the fact that both T and DHT enhanced the transcript levels of *Drd2* and *Adcy5*. A similar pattern after ORX and hormone replacement was reported in a recent study in adolescent rats^28^. Since we show that changes in wheel running occurred very early after ORX, it is possible that we have “missed” early androgen-regulated changes in gene expression. Another possibility is that T acts primarily on the DA system by regulating the availability of extracellular DA^44–46^. Remarkably, ORX not only influences the basal concentrations of extracellular DA but also its release upon stimulation^46^. We hypothesize that ORX mice display a hypo-dopaminergic state and that treatment with T rescues their wheel running activity by restoring the basal dopaminergic tone. In contrast, T might restrain the enhanced release of DA in target brain regions of ORX animals after amphetamine challenge, thus attenuating the resultant locomotor response. Our results indicate that T restores the sensitivity to amphetamine in male mice through ERs-dependent mechanisms, since DHT was ineffective. These findings are supported by a recent study in rats showing similar actions for ORX and T after amphetamine challenge^47^.

Androgens are known to exert quick-acting effects on male behavior^29,48^. Intranasal administration is particularly effective in delivering T to the brain in mice^49^. However, in our conditions, a short-exposure to intranasal T did not attenuate the hyperlocomotion response in ORX mice after amphetamine challenge. Thus, the results do not support a role for T acting on the DA system through either non-genomic or rapid genomic mechanisms.

Finally, we tested if the selective antagonism of DA receptors could significantly prevent the wheel running induced by T. Addition of low doses of the DR2 antagonist haloperidol to the drinking water induced a reduction in wheel running that was far more pronounced in ORX + T- than ORX + placebo-treated mice. In a similar pattern, treatment with the DR2 antagonist L-741,626 reduced by almost 50% the distance ran by ORX + T-treated mice. This 741,626 regimen however was not effective decreasing wheel running in ORX + placebo-treated animals or in Balb/cJ female mice^50^. Therefore, it is unlikely that the doses of 741,626 used here altered *per se* the physical ability of mice to run in the wheel. We conclude that DR2-dependent pathways are implicated in the T-induced stimulation of wheel running.

As discussed above, a limitation of our study is that we could not identify the precise mechanisms through which T modulates the DA system to increase wheel running. In addition, due to the complexity of the biology of DA receptors interactions, we cannot rule out the contribution of DR1, despite the doses of oral SCH23390 used here exerted biological effects *in vivo*^50^. Finally, other neurotransmitter systems, like those involved in energy regulation, are likely involved in additionally mediating the neuroendocrine effects of T on wheel running behavior.

In summary, our study reveals that T deficiency impairs physical activity in male mice manifestly, and that this effect is a purely central phenomenon and not the result of a muscle dysfunction. We also provide a novel illustration that free T acts as prohormone for E2 in the brain to regulate wheel running via DA-dependent pathways. Clinically, the impact of T actions on physical activity could represent an additional benefit of T replacement therapy in ageing men with sarcopenia, dysmobility and low T.

## Methods

### Mice

Male C57BL/6J mice were obtained from the KU Leuven animal facility, prior confirmation of genetic background purity by JAX SNP panels. Generation of male C57BL/6J mice with a ubiquitous knockout of the androgen receptor (ARKO) has been previously described^51^. Male ARKO mice display a complete androgen insensitivity syndrome with smaller gonads located in the abdominal region^51^. Heterozygous SHBG_(4.3kb)_^+/−^ mice were a generous gift from prof. GL. Hammond (University of British Columbia, Vancouver, B.C, Canada). Male mice heterozygous for a cyclization recombinase driven by the MyoD-iCre (generously donated by dr. D. Goldhamer, University of Connecticut, Storss, CT, USA) were bred with female mice heterozygous for a floxed AR exon 2 allele to generate male conditional satellite-cell lineage ARKO (satARKO, AR^flox/Y^Cre^+/−^) and male control (pool of AR^wt/Y^Cre^−/−^, AR^flox/Y^Cre^−/−^ and AR^wt/Y^Cre^+/−^ genotypes) mice, as described^27^.

Mice were group-housed at 20 °C with a 12h-dark/light cycle with *ad libitum* access to food and water, according to our institutional guidelines. For *ex vivo* analyses, animals were euthanized by pentobarbital anesthesia followed by cardiac puncture. Tissues were dissected and wet weight measured.

### Surgical procedures

At 16 weeks of age, both male C57BL/6J and ARKO mice and their male wild type (WT) littermates underwent ORX (abdominal approach) under isoflurane anaesthesia followed by buprenorphine analgesia (60 µg/kg SC). Implants of medical-grade silicone tubing (Silclear®, Degania Medical, Degania, Israel) sealed with medical adhesive silicone (Silastic®, Biesterfeld, Germany) were implanted in the nuchal region, either empty or filled with T or DHT (both from Sigma-Aldrich, St. Louis, MO, USA). The same surgical procedure except for the removal of the testis or gonads was conducted in sham animals, which received an empty implant. Also at 16 weeks of age, both male SHBG-Tg and satARKO mice and their respective male WT littermates were ORX and replaced with a T implant. Under these conditions, we have previously demonstrated that male SHBG-Tg mice display a mild hypogonadism phenotype, characterized by a decrease in free T serum levels and a reduction in the weights of seminal vesicles and levator ani/bulbocavernosus (LA/BC) muscle complex^21^.

### Wheel running

Following 2 days of post-surgery recovery, animals were introduced into individual cages equipped with computerized running wheels (Linton Instrumentation Inc., Diss, Norfolk, UK). Revolutions were measured electronically in 10-min bins of activity for 19 days and afterwards the running distance was calculated.

### Treatment with dopamine antagonists

Haloperidol (Haldol®, Janssen-Cilag, Berchem, Belgium) and SCH23390 (Tocris Bioscience, Ellisville, MO, USA) were dissolved in distilled water and then administered in the drinking water at doses of 0, 0.15 and 0.30, and 0, 0.05 and 0.1 mg/kg-day, respectively. The concentrations of haloperidol and SCH23390 were calculated taking into account the average daily water consumption and weight of mice. Bottles were wrapped in aluminum foil and the drugged water was renewed on a daily basis. Prior to drug exposure, wheel running at the plateau phase was recorded for three consecutive days and taken as baseline. L-741,626 (Tocris Bioscience) was first dissolved in ethanol and then diluted with distilled water (final ethanol concentration 6%). The animals were accustomed to handling and i.p. injections with vehicle for 7 days before exposure to L-741,626 (1, 2, 5 and 10 mg/kg). The last three days of the habituation period were used as baseline.

### Treatment with enzalutamide

Enzalutamide (Sequoia Research Products, Pangbourne, United Kingdom) was prepared in 1% carboxymethyl cellulose, 0.5% Tween 80, and 5% dimethylsulfoxide^52^. Wheel running was recorded for three days during which animals were treated daily with vehicle, and then 3 days per dose of enzalutamide (30 and 60 mg/Kg, p.o.).

### Home-cage activity

A new group of animals with non-access to a wheel was used, as to avoid the confounding effects of previous running^53^. Mice were housed individually in 20 × 30 cm^2^ transparent cages located between three photo beams and activity was measured as beam crossings for each 30 min, during a 23 h interval.

### Locomotion response to amphetamine

Following a 180-min habituation period in the activity cage, mice received an i.p. injection of vehicle (saline, 10 mL/Kg) and were monitored for another 60 min. Afterwards, the procedure was repeated but, instead of saline, animals were challenged with amphetamine (3 mg/kg; Tocris Bioscience). The hyperlocomotion response to the drug was recorded for 180 min. After a three week wash-out period, the experiment was repeated with a lower dose of amphetamine (0.75 mg/kg).

In another set of experiments, ORX + placebo-treated mice were subjected to the former procedure but, prior to the challenge with 3 mg/kg amphetamine, they received either intranasal T (2 mg/kg, 12 µL per mouse) or vehicle (sesame oil). A subgroup of animals was euthanized 25 min after T or vehicle administration and the levels of T in serum and brain extracts were determined by liquid chromatography tandem mass spectrometry (LC-MS/MS).

### Elevated plus maze

The apparatus consisted of an elevated (30 cm) maze with two closed (21 cm long × 5 cm wide) and two open arms making the shape of a cross. Mice were placed at the center of the maze and were allowed to move freely for 10 min. The number of arm entries was recorded by four infrared beams.

### Morris-type water maze

The test included two probe trials, in which the hidden platform was removed from the pool, after each five days of training. The learning curves (using swim path length to reach the hidden platform) were determined over 10 days of training. For the probe trials, the time spent in each quadrant was calculated.

### Grip strength

Total-limb maximal grip strength was evaluated by means of a grid connected to an isometric force transducer (Chatillon DFIS-2 Digital Force Gauche, Ametek, Paoli, PA, USA). Mice grasping a metal grip with all limbs were pulled horizontally by their tails until they lost their grip. Measurements were registered in Newtons (N) and the result was set as the average of six attempts.

### Rotarod test

General motor performance was assessed using an accelerating rotarod (Ugo Basile, Comerio, Italy) rotating from 4 to 40 rpm on 5 min ramp duration. Each mouse was subjected to six sessions without prior training and the latency to fall was recorded for each trial.

### Neuron counts

Paraformaldehyde-fixed lumbosacral spinal cord tissue samples were processed routinely for paraffin embedding, and 20-*μ*m sections were obtained for Nissl staining. All images were acquired and analyzed using a Zeiss Axiovert microscope. Cresyl violet positive neurons located in the ventral horn, with a soma area over 250 μm^2^ and a clearly defined nucleus were included in the counts.

### Electrophysiology

Mice were anaesthetized under a 2.5% oxygen flow containing 1.5–2% isoflurane. Nerve conduction studies were performed using sub-dermal needle electrodes (Technomed Europe, Maastricht, Netherlands) and a Natus UltraPro S100 (Natus Medical Incorporated, San Carlos, CA, USA). Compound muscle action potentials (CMAPs), were determined by measuring the response at the gastrocnemius muscle after stimulation occurred at the level of the sciatic notch.

### DXA

Lean and fat mass were analyzed *in vivo* using the PIXImus mouse densitometer (Lunar Corp) with an ultrahigh resolution (0.18 × 0.18 pixels, 1.6 line pairs/mm) and software version 1.45.

### Liquid chromatography- tandem mass spectrometry

Sex steroid concentrations were measured using LC-MS/MS. Prior to LC-MS/MS analysis, brain tissues were homogenized and extracted with diethyl ether, as previously described^54^. Total T and E2 in both serum and brain tissue extracts were measured at the University Hospitals Leuven by LC-MS/MS without derivatization using a two-dimensional chromatography system and an AB/Sciex QTrap 5500 tandem mass spectrometer in atmospheric pressure chemical ionization positive and electrospray ionization negative ion mode, respectively.

### Analysis of the total monoamine/metabolite content

The total noradrenaline (NAD), dopamine (DA) and serotonin (5-HT) content as well as their metabolites 3,4-dihydroxyphenylacetic acid (DOPAC), homovanillic acid (HVA) and 5-hydroxyindolacetic acid (5-HIAA) in the striatum were measured based on previously reported methods^55^. In summary, after weighing striatal tissue, 190 µL of an antioxidant solution (0.1 M acetic acid, 3.3 mM L-cysteine, 0.27 mM Na2EDTA and 0.0125 mM ascorbic acid) and 10 µL of an internal standard solution (3,4-dihydroxybenzylamine solution 1 µg/mL in antioxidant) were added to the tissue. After homogenization, the samples were centrifuged (20 min, 9500 g, 4 °C). The supernatant was diluted 5-fold in 0.5 M acetic acid and 20 µL was injected automatically on a reversed phase liquid chromatography system (autosampler ASI-100 and HPLC pump P680 A HPG/2, Dionex, Amsterdam, The Netherlands) with electrochemical detection (potential = +700 mV) (Amperometric Detector LC-4C, BAS). The separation was achieved using a narrowbore C18 column (Alltech®, AlltimaTM, 5 µm, 150 × 2.1 mm, Grace, Deerfield, IL, USA). The mobile phase buffer contained 0.1 M sodium acetate, 20 mM citric acid, 1 mM sodium octane sulfonic acid, 1 mM dibutylamine and 0.1 mM Na2EDTA adjusted to pH 3.7 (mobile phase composition: 97 buffer/3 methanol (v/v)). Tissue concentration was expressed as ng monoamine (or metabolite)/g wet tissue (ng/g).

### Quantitative PCR

Total RNA was extracted from tissue samples by homogenization in Trizol reagent (Invitrogen, Carlsbad, CA, USA) followed by isopropanol precipitation. For cDNA synthesis, a superscript II RNaseH– reverse transcriptase kit was used (Invitrogen). The primer sequences are described in Table 1. The PCR mixtures (10 µl) contained 5 µl Fast SYBR green Master Mix (Applied Biosystems, Foster City, CA, USA) and 0.03 µM of each primer. For quantification of gene expression, the StepOnePlus™ sequence detector PCR detection system (Applied Biosystems) was used. The housekeeping gene hypoxanthine guanine phosphoribosyl transferase (*Hprt*) served as an endogenous control and expression levels were analyzed by the 2−ΔΔCT method.

**Table 1:** Primers used for qRT-PCR.

Gene		Sequence (5′ → 3′)
*Adcy5*	Fwd	AAGATCCTCGGGGATTGTTACT
	Rev	CTCCCGGACCAACGAGATG
*Comt*	Fwd	CTTCCTGGCGTATGTGAGGG
	Rev	CAAGCCGTCCACCACTTTCAT
*Dlg4*	Fwd	TCTGTGCGAGAGGTAGCAGA
	Rev	AAGCACTCCGTGAACTCCTG
*Drd1*	Fwd	ATGGCTCCTAACACTTCTACCA
	Rev	GGGTATTCCCTAAGAGAGTGGAC
*Drd2*	Fwd	CAAGCGCCGAGTTACTGTCAT
	Rev	ATGGAGGAGTAGACCACGAAG
*Gnal*	Fwd	GCCAACAAAAAGATCGAGAAGC
	Rev	GTTGAAGCCATTGACGTGCAG
*Hprt*	Fwd	TTATCAGACTGAAGAGCTACTGTAATGATC
	Rev	TTACCAGTGTCAATTATATCTTCAACAATC
	Probe	TGAGAGATCATCTCCACCAATAACTTTTATGTCCC
*Maoa*	Fwd	GCCCAGTATCACAGGCCAC
	Rev	CGGGCTTCCAGAACCAAGA
*Maob*	Fwd	ATGAGCAACAAAAGCGATGTGA
	Rev	TCCTAATTGTGTAAGTCCTGCCT
*Nefm*	Fwd	AAGTGGGAAATGGCTCGTCA
	Rev	TGGTCTCTTCCCCCTCTAGG

### Statistics

Statistical analysis was performed using Graphpad Prism v6.05. Student t-test and one- way ANOVA followed by Bonferroni’s post-hoc test were used to analyze differences between two groups or more, respectively. When two independent variables were included in the analysis, two-way ANOVA was performed. Evolution of groups over time was analyzed with repeated measures ANOVA. All statistical tests were performed two-tailed. Data are expressed as mean ± SEM and P values < 0.05 were considered statistically significant.

### Study approval

All experiments involving animals were performed in accordance with relevant guidelines and regulations and were conducted with approval of the KU Leuven ethical committee (P041/2014).

## Electronic supplementary material

Supplementary material
