# Supplementary material for: Testosterone boosts physical activity in male mice via dopaminergic pathways
Source: Sci Rep. 2018 Jan 17;8:957. doi: 10.1038/s41598-017-19104-0 (PMC5772634; doi:10.1038/s41598-017-19104-0)
Supplement: Supplementary file 1 — Supplementary material [file 41598_2017_19104_MOESM1_ESM.pdf]

Testosterone boosts physical activity in male mice via dopaminergic pathways

Ferran Jardí,[1] Michaël R. Laurent,[2,3] Nari Kim, [1] Rougin Khalil,[1] Dimitri De Bundel,[4] Ann Van Eeckhaut,[4] Lawrence Van Helleputte,[5] Ludo Deboel,[1] Vanessa Dubois,[2,£] Dieter Schollaert,[1] Brigitte Decallonne,[1] Geert Carmeliet,[1] Ludo Van den Bosch,[5] Rudi D'Hooge,[6] Frank Claessens,[2] and Dirk Vanderschueren[1]

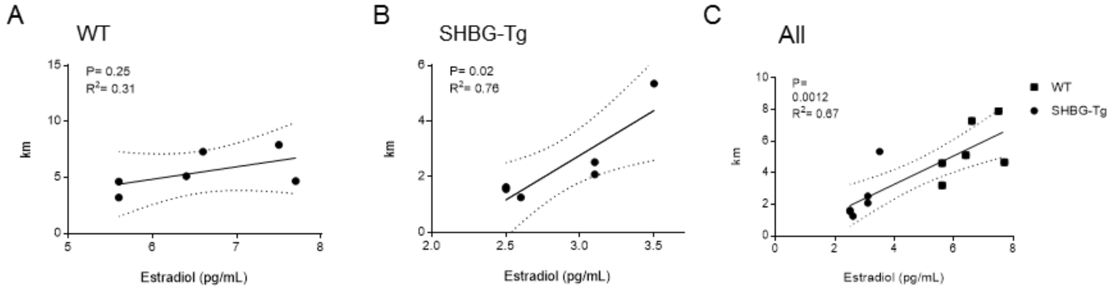

Supplementary Figure 1. Correlations of E2 concentrations in brain homogenates with average distance run per day in WT (A), SHBG-Tg mice (B) and both genotypes (C). Note that in C squares represent WT and circles SHBG-Tg. Correlations were examined using Pearson's r and linear regression. The 95% confidence band of the linear regression is shown as a dotted line.

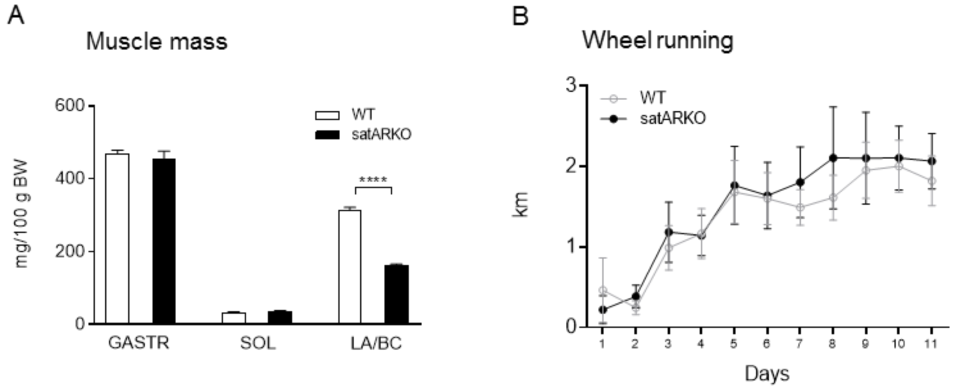

Supplementary Figure 2. Myogenic AR does not drive T effects on wheel running activity. GASTR, SOL and LA/BC weights (A) and time course of daily wheel running activity over a period of 11 days (B) in satARKO and WT mice, both treated with T following ORX. Data in panel A were analyzed using Student t-test and those in panels B were analyzed by two-way repeated measures ANOVA. All data are mean ± SEM, 4-8 animals per group. Statistical significance levels: **** = P< 0.0001. GASTR gastrocnemius, SOL soleus, LA/BC levator ani/bulbocavernosus.

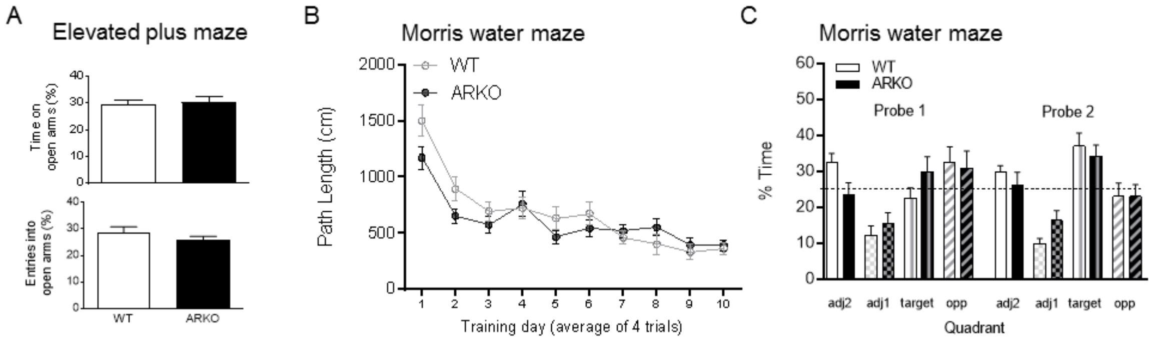

Supplementary Figure 3. A, Elevated plus maze test. Percentage of entries and time spent in open arms by WT and ARKO mice. B and C, Morris water maze test. Path length to the platform over 10 days of training (B) and percentage of time spent in the target quadrant during the probe trial (C) in WT and ARKO mice. The dotted line indicates the chance level (25%). Data are presented as mean ± SEM. In A, n=17-20 animals per group; in B and C, n=15 animals per group. Adj adjacent, opp opposite.

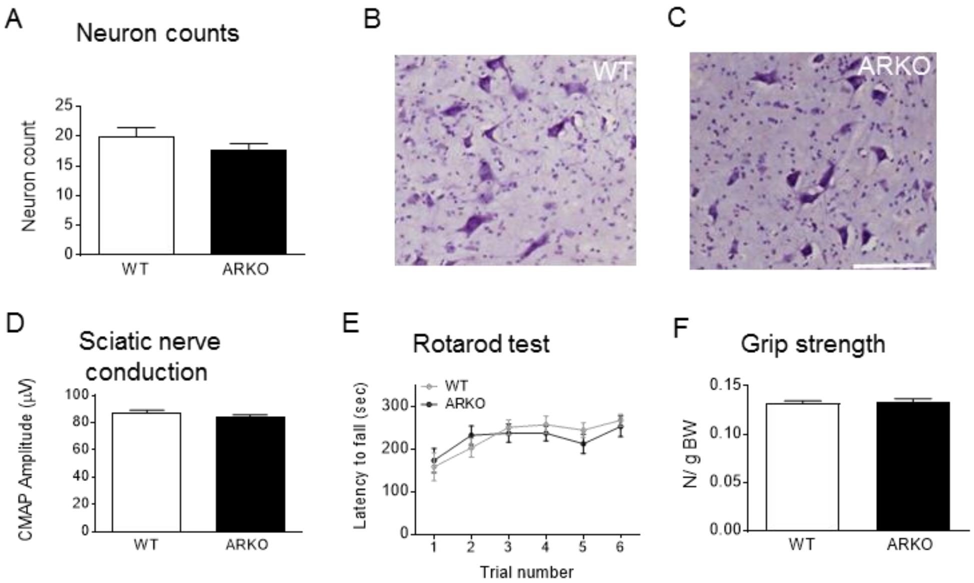

Supplementary Figure 4. A, Motor neuron counts in the ventral horn of the lumbar spinal cord in WT and ARKO mice. Photomicrographs showing Nissl-stained lumbar spinal cord sections from a WT (B) and an ARKO mouse (C). Scale bar represents 200 µm. Mean sciatic CMAP amplitudes (D), rotarod time course for latency to fall over 6 trials (E) and grip strength (F) in WT and ARKO mice. Data are presented as mean ± SEM. In A, n=7-9 animals per group; in D and E, n=8-9 animals per group; in F, n=8 animals per group. CMAP compound muscle action potential.

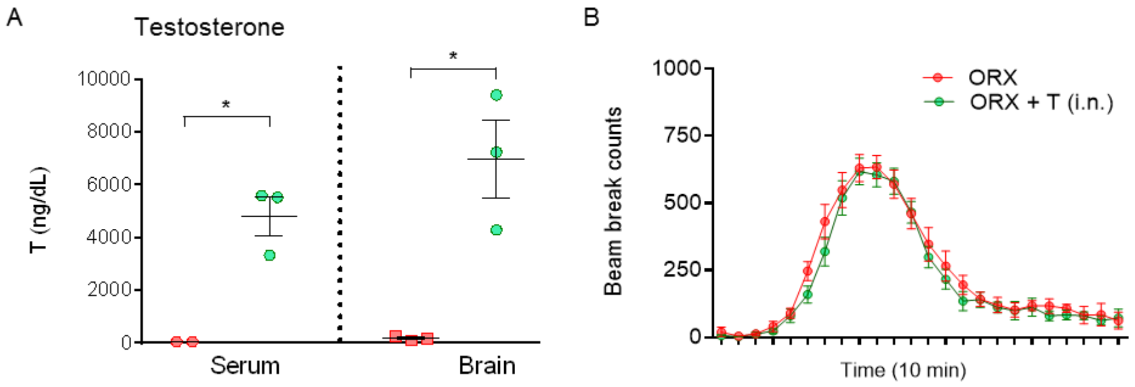

Supplementary Figure 5. A, Concentrations of T in serum and brain homogenates of ORX mice 25 min after an intranasal administration of vehicle or T. B, Time course of locomotor-induced behavior after i.p. injection with 3 mg/kg amphetamine in ORX mice pre-treated with intranasal vehicle or T. Data in panel A were analyzed using Student t-test and those in panel B were analyzed by two-way repeated measures ANOVA. Data are presented as mean ± SEM. In A, n=2-3 animals per group; in B, n=6 animals per group. Statistical significance levels: *, = P<0.05.
